# The “*Journal of Functional Morphology and Kinesiology*” Journal Club Series: Highlights on Recent Papers in Exercise-Induced Immune Response

**DOI:** 10.3390/jfmk3030042

**Published:** 2018-07-25

**Authors:** Francesca Luchetti, Maria Gemma Nasoni, Elisabetta Falcieri, Alexandrina Ferreira Mendes

**Affiliations:** 1 Department of Biomolecular Sciences, University of Urbino Carlo Bo, 61029 Urbino, Italy; 2 Faculty of Pharmacy and Centre for Neuroscience and Cell Biology, University of Coimbra, 3004-504 Coimbra, Portugal

## Abstract

We are glad to introduce the ninth Journal Club. This edition is focused on several relevant studies published in the last few years in the field of Exercise-Induced Immune Response, chosen by our Editorial Board members and their colleagues. We hope to stimulate your curiosity in this field and to share with you the passion for sport seen also from the scientific point of view. The Editorial Board members wish you an inspiring lecture.

## 1. Introduction

In the last few years, exhaustive papers highlighted how regular physical exercise extends life expectancy and reduces the risk of chronic diseases. In particular, these papers have widened the ways in which we study physical exercise, not only for the aim of improving sports performance but also highlighting the importance of physical exercise in daily life. The skeletal muscle system represents approximately 40% of the body’s weight and it is composed of multinucleated fibres and satellite cells. The satellite cells have important roles in the regeneration of muscle, but to date there is still much we do not know about their function. The proper function of a tissue is also the result of communication among tissues. The proper function of muscle tissue is largely influenced by immune cells. In particular, immune cells are able to drive the proliferation or differentiation of satellite cells, thus playing a key role in the homeostasis of muscle tissue.

## 2. Recent Papers Regarding Exercise-Induced Immune Response

### 2.1. The Interplay between Satellite and Immune Cells in the Regeneration of Muscle

#### 2.1.1. Highlight by F. Luchetti, M.G. Nasoni, E. Falcieri

There is a growing body of epidemiological evidence that regular physical exercise extends life expectancy and reduces the risk of chronic diseases. However, physical exercise, in particular, exercise consisting of highly strenuous bouts such as a marathon or ultramarathon, could cause a muscle damage and could transiently induce a perturbation of the immune system. The same is true for the sarcopenia in the elderly. The skeletal muscle system represents approximately 40% of an average individual’s body weight and it is composed of multinucleated fibres and satellite cells. Muscle satellite cells (mSCs) are examples of adult stem cells for skeletal muscles.

The capability of skeletal muscles of repairing themselves is due to the SCs [1,2]. These cells are located between the basal lamina and the sarcolemma. The primary function of these cells is to mediate muscle growth and repair in the postnatal life [3].

Several authors have tried to identify surface and intracellular markers in order to unequivocally identify this population. However, recent papers have demonstrated that the characteristics of SCs depend on muscle type. Ono et al. [4] reported that the gene expression as well as the abilities of stem cells are different when observed in the extensor digitorum longus or in the masseter muscle. However, most of the papers have identified Pax7 as a transcription factor whose expression is critical for identifying the SC population. Pax3 is also expressed by SC but only in a subset of SCs of some but not all muscles. In response to injury, SCs proliferate and their Pax7-positive daughter cells either differentiate, by migrating through the sarcolemma and fusing with existing muscle fibres during the growth and regeneration of muscle [5,6], or they commit to a program of self-renewal.

#### 2.1.2. How Do the Immune Cells Drive the Fate of Satellite Cells?

Adult skeletal muscles contain different types of resident leukocytes, in particular, mast cells and macrophages. In the presence of a damaged muscle, the immune cells are able to release several cytokines, such as interleukin 6 (IL-6), TNF-α, and tryptase; at a low physiological concentration these cytokines promote activation and proliferation of SCs [7]. The initial burst of cytokines and chemokines produced by resident leukocytes leads to the rapid attraction of circulating granulocytes to the damaged area. The neutrophils are involved in the clearance of cellular debris, moreover the neutrophils release the chemokines such as MIP-1α and MCP-1 that favour the recruitment of monocytes. Arnold and coworkers [8] demonstrated that the population of monocytes divides itself into two categories: Ly6C^+^ and Ly6C^−^, where the Ly6C^+^ monocytes promote the release of proinflammatory cytokines whereas Ly6C^−^ monocytes express high levels of anti-inflammatory molecules and growth factors. These two populations exert opposite effects on SCs; the Ly6C^+^ monocytes promote the proliferation of SCs, whereas the Ly6C^−^ population has an opposite effect and stimulates the differentiation of SCs. However, these opposite effects are very important to maintain the balance between the proliferation and differentiation of myogenic cells. It is well known that when the monocytes invade tissues they begin to differentiate in macrophages. Also, macrophages can be divided into several subtypes during muscle regeneration. The macrophages are classified as M1 and M2. M1 macrophages have been found close to proliferating myogenic cells, whereas M2 macrophages interact with differentiating myocytes [9]. The ability of macrophages to induce myogenic effects is due to the physical contact and paracrine signalling. The direct contact with SCs is mediated by adhesion molecules such as VCAM-VLA4; this interaction allows macrophages to inhibit apoptosis and promote cell survival. Sonnet and coworkers [10] investigated this phenomenon in detail. In fact, they have demonstrated the ability of macrophages to reduce the number of TUNEL-positive apoptotic myogenic cells during post-injury muscle regeneration in mice. In addition, macrophages deliver anti-apoptotic signals through all four adhesion systems, i.e., VCAM-1-VLA-4, ICAM-1-LFA-1, PECAM-1-PECAM-1, and CX3CL1-CX3CR1. On the contrary, the paracrine signalling is mediated by IL-1β, IL-6, and TNF-α—these cytokines are released by M1 macrophages and are able to promote the proliferation of myogenic cells; on the other hand, IL-4 and IGF-1 secreted by M2 macrophages can induce the differentiation [11]. Recently, an excellent paper by Nie and coworkers [12] demonstrated that miRNA can affect muscle regeneration by modulating myeloid cells in injured muscle. They showed that miR-155-deficient mice exhibit delayed muscle regeneration and an aberrant macrophage activation leading to an unbalance between the expression of pro- and anti-inflammatory cytokines. In conclusion, the studies cited above demonstrated that the sum of interactions among systems is critical for maintaining the pool of SCs.

### 2.2. Acute Aerobic Exercise Induces a Preferential Mobilisation of Plasmacytoid Dendritic Cells into the Peripheral Blood in Humans

#### Highlight by Alexandrina Ferreira Mendes

Accumulating evidence indicates that cells of the immune system are sensitive to physical activity and exercise. The most well-established change occurs in response to acute aerobic exercise and involves increased recruitment of immune cells to the blood stream followed by a quick decline [13]. This redistribution of leukocytes to peripheral tissues seems to be especially significant for subsets with characteristics of effector cells, which has been interpreted as a conserved evolutionary response to enhance immune surveillance [14]. Cells of lymphoid origin, namely T lymphocytes and natural killer cells, have been most extensively studied because these are the cells most responsive to exercise [15,16,17,18,19]. Less attention has been devoted to the impact of exercise on myeloid lineage cells [19,20,21] and only a few studies have examined its role on dendritic cells (DCs), despite their critical role in antigen presentation and regulation of the immune responses. The study by Brown and collaborators [22] addresses this question by quantifying the number of DCs mobilized to the blood stream by acute aerobic exercise of moderate intensity and by phenotypically characterizing different DC subpopulations by flow cytometry. DCs were identified as lineage negative (that is, negative for markers of both lymphoid and myeloid cells, namely CD3, CD19, CD20, CD14, and CD56) HLA-DR positive CD303 negative cells (lineage^−^ HLA-DR^+^ CD303^−^). The total number of DCs in peripheral blood increased by 150% relative to resting pre-exercise numbers, during the last minute of a 20-min duration cycling exercise. Both plasmacytoid (CD303^+^) and myeloid (CD303^−^) DCs contributed to the increase, but the mean increase in plasmacytoid DC number was greater than that observed for myeloid DCs. Thirty minutes after completion of the exercise, the numbers of both DC subsets in the peripheral blood decreased but remained above pre-exercise numbers. Since plasmacytoid DCs produce type 1 interferons and have a more inflammatory profile and migration capacity than myeloid DCs, their preferential increase in response to exercise may represent an adaptive response to increase the ability to fight infections, especially caused by viruses. On the other hand, myeloid DCs, which also increased in response to exercise, have a major role in promoting T cell activation and differentiation [23,24], suggesting that exercise can also improve effector T cell responses. Then, to differentiate myeloid DC subsets, the authors used antibodies against CD1c, which characterizes DCs capable of stimulating CD4^+^ T cells, and CD141, involved in the cross-presentation of antigens to CD8^+^ T cells, which are especially relevant in anti-tumour activity. The four subsets defined by these markers increased with exercise, with the largest increase occurring with the CD1c^−^ CD141^−^ subset and the smallest with the CD1c^–^ CD141^+^ myeloid DCs. Furthermore, the numbers of DCs that were or were not expressing CD205, a cell surface receptor involved in the recognition of necrotic and apoptotic cells, and CD209, involved in adhesion, migration, and antigen presentation, were also evaluated. The results showed that although not very abundant, CD205^−^ and CD209^−^ DCs increased proportionately more in response to exercise than their positive counterparts, suggesting that exercise preferentially recruits immature DCs. In conclusion, this study shows that DCs are exercise-responsive and that distinct subsets are differentially mobilized by acute exercise. Besides considerations surrounding their physiological importance, these findings may be especially relevant to the optimization of protocols for harvesting DCs from the blood for use in DC-based immunotherapies, which are already in clinical use for cancer therapies. Although further studies are required to fully elucidate the kinetics of DC mobilization to peripheral blood and redistribution to tissues in response to exercise, namely by identifying the best exercise protocol in terms of intensity, type, and duration to increase the yield in total and specific DC populations, the present study contributes to strengthen the potential role of exercise as a simple inexpensive strategy to improve DC collection for immunotherapies. Moreover, other factors, like gender and age, also affect the number and functions of immune cells in response to exercise [19,25] and may, therefore, also modulate the effects of exercise on the mobilization of total DCs and subpopulations, which deserves further investigation.
